# In-utero and newborn factors and thyroid cancer incidence in adult women in the Sister Study cohort

**DOI:** 10.1038/s41416-025-03004-6

**Published:** 2025-04-09

**Authors:** Thi-Van-Trinh Tran, Katie M. O’Brien, Rebecca Troisi, Dale P. Sandler, Cari M. Kitahara

**Affiliations:** 1 Radiation Epidemiology Branch, Division of Cancer Epidemiology and Genetics, National Cancer Institute, National Institutes of Health, 9609 Medical Center Drive, Bethesda, MD 20892-9778 USA; 2 Epidemiology Branch, National Institute of Environmental Health Sciences, Research Triangle Park, Durham, NC USA; 3 Trans-Divisional Research Program, Division of Cancer Epidemiology and Genetics, National Cancer Institute, National Institutes of Health, 9609 Medical Center Drive, Bethesda, MD 20892-9778 USA

**Keywords:** Risk factors, Cancer epidemiology

## Abstract

**Background:**

Thyroid cancer is diagnosed at relatively young ages compared to other adult cancers, for reasons that remain unclear. Our study aimed to investigate associations of in-utero and newborn characteristics with differentiated thyroid cancer (DTC) incidence in adult women.

**Methods:**

From the U.S. nationwide Sister Study cohort, we included 47,913 cancer-free women at baseline (2003–2009). We assessed associations of participants’ in-utero and newborn characteristics and DTC during follow-up using Cox regression models adjusted for attained age (timescale) and race/ethnicity.

**Results:**

During follow-up (median = 13.1 years), 239 incident DTC cases were identified. Higher DTC incidence was associated with maternal pre-pregnancy or gestational diabetes (hazard ratio [HR] = 2.36, 95%CI = 0.97–5.74, 5 affected cases), gestational hypertension or hypertension-related disorders (HR = 1.99, 95%CI = 1.20–3.32, 16 affected cases), and higher birth weight (HR per kg=1.24, 95%CI = 0.95–1.60). Births occurring at least two weeks before the due date were associated with lower DTC incidence (HR = 0.47, 95%CI = 0.23–0.97, 8 affected cases). In a model simultaneously adjusted for all these factors, all exposures remained associated with DTC incidence. We observed no associations for other in-utero and newborn characteristics.

**Conclusions:**

These findings contribute to a growing body of evidence that in-utero exposures related to maternal metabolic abnormalities may influence thyroid cancer risk later in life.

## Introduction

Thyroid cancer has emerged as the fifth most common cancer diagnosed among women globally, with most cases classified as differentiated thyroid cancer (DTC) [1]. Yet, the etiology of thyroid cancer remains largely unknown, with obesity and childhood exposure to ionizing radiation among the few established modifiable risk factors [2]. The disease is diagnosed at relatively young ages compared to most other adulthood cancers [2], suggesting a potential etiologic role of early-life exposures [3, 4].

Throughout gestation, the developing thyroid is highly susceptible to endogenous (e.g., maternal iodine deficiency, thyroid dysfunction, diabetes, fluctuations in maternal and placental estrogen levels) [5–9] and exogenous (e.g., cigarette smoking, endocrine-disrupting chemicals [EDCs]) stressors. These factors may disrupt fetal thyroid function [10, 11], expose the fetus to elevated levels of growth factors [12] and sex steroid hormones [13], which have been suggested to contribute to thyroid cancer development in adulthood [3, 4, 14–16]. Some epidemiologic studies have suggested positive associations for factors reflecting fetal hormone exposures, including maternal and congenital thyroid disorders [17], higher birth weight [17–19], postpartum hemorrhage [17], and hyperemesis gravidarum [20], lending some support to the hypothesis that the gestational period is a susceptible exposure window in thyroid carcinogenesis. However, evidence on the impact of in-utero and birth characteristics on subsequent risk of thyroid cancer remains scarce.

To contribute to the limited research on this topic, we prospectively evaluated associations of a wide range of self-reported in-utero and newborn factors with DTC incidence in the Sister Study, a large U.S. nationwide cohort including more than 50,000 adult women who had a sister with a breast cancer diagnosis but were themselves breast cancer-free at enrollment.

## Methods

### Study sample

The study design, data collection, and outcome measurements of the Sister Study cohort have been previously described [21]. Briefly, between 2003 and 2009, the Sister Study recruited women aged 35–74 residing in the United States (including Puerto Rico) who had a sister diagnosed with breast cancer but no prior diagnosis themselves. At baseline, participants provided detailed information on early-life, demographic, medical, and lifestyle factors through computer-assisted telephone interviews and written questionnaires. Anthropometric measurements were also collected during in-person examinations. Detailed follow-up questionnaires were sent every 2–3 years, with response rates consistently exceeding 85% [22]. The study is approved annually by the National Institutes of Health Institutional Review Board. All participants provided written informed consent. Data are complete through mid-September 2021 (data release 11.1, https://sisterstudy.niehs.nih.gov/English/cohort-over.htm, https://sisterstudy.niehs.nih.gov/English/enroll-data.htm#SAQS).

**Fig. 1:**
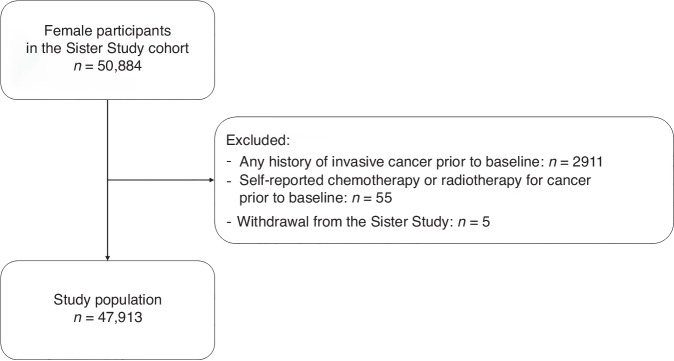
Flowchart of selection of the study population. Flowchart showing number of included individuals and exclusions.

Of the 50,884 participants, we excluded those who had a history of any invasive cancer (*n* = 2911) or reported any chemotherapy or radiotherapy for cancer (*n* = 55) before baseline, and those who withdrew from the study (*n* = 5). After exclusions, there were 47,913 individuals in our study sample (Fig. 1).

### Outcome definition

Thyroid cancer diagnoses were identified through self-report and when available, confirmed using medical records or pathology reports. By the end of follow-up in mid-September 2021, 252 cases had been reported, of which 187 were verified through medical records or pathology reports, and one additional case was confirmed via the National Death Index, death certification, or next of kin, resulting in 188 (74.6%) confirmed cases. Agreement between self-report and confirmed thyroid cancer diagnosis was excellent (positive predictive value >90%). For the current analysis, the case definition for DTC (*n* = 239) included all self-reported cases excluding those with the following histologic types: poorly differentiated thyroid carcinoma (confirmed with pathology reports; *n* = 5), anaplastic thyroid carcinoma (histology code: 8021; *n* = 1), medullary thyroid carcinoma (histology codes: 8346, 8347, 8510, *n* = 5), and indeterminate (histology code: 8265, 9084, *n* = 2). DTC cases with confirmed histology codes (*n* = 174) were further classified according to the following histologic types: papillary thyroid carcinomas (histology codes: 8050, 8260, 8340-8344, 8350, 8450-8460, *n* = 164), follicular thyroid carcinomas (histology codes: 8290, 8330-8335, n = 7), and unspecified carcinomas and neoplasms (histology code: 8000, 8010, *n* = 3).

### Exposure definition

Using the baseline self-administered family history questionnaires, we ascertained information on participants’ perinatal exposures, including maternal, paternal, and birth characteristics. Characteristics of the participants’ mothers before and during pregnancy included pre-pregnancy or gestational diabetes, gestational hypertension or hypertension-related disorders (i.e., pre-eclampsia, eclampsia, and toxemia), morning sickness with vomiting, first-hand or household secondhand smoking during pregnancy, time since last pregnancy, and age at delivery. Information on the participants’ fathers characteristics included first-hand smoking during three months before conception, and age at delivery. Participants’ own birth and postpartum characteristics included birth weight, birth order, singleton versus multiple birth, gestational age at birth, whether they were breastfed, and duration of breastfeeding. For all of these, we considered responses of “definitely” and “probably” as exposed and categorized “probably not” and “definitely not” as unexposed. For birth weight analyses, women were asked to provide their birth weight, which we classified into clinically relevant categories: <2500 g, 2500–3999 g, and ≥4000 g. Women who did not specify their birth weight but reported it being less than 5 pounds (2268 g) were categorized into the <2500 g category. Missing data were classified using missing indicator variables.

### Baseline covariates

In-person measurements of weight and height at baseline were used to calculate body mass index (BMI, weight in kilograms divided by the square of height in meters). Information on other characteristics, including age, self-identified race/ethnicity (Non-Hispanic White, Non-Hispanic Black, Hispanic, Non-Hispanic all other races, Unknown), personal attained educational level, personal history of benign thyroid disease, and household annual income were assessed during the computer-assisted telephone interview. Individuals were considered to have a history of benign thyroid disease if they reported a diagnosis of hyperthyroidism, hypothyroidism, non-toxic goiter/thyroid nodules, or any other thyroid conditions, or if they ever self-reported use of thyroid hormone substitutes, antithyroid, or iodine/iodide substances for medical purposes or supplements for thyroid function.

### Statistical analyses

Follow-up time was calculated from age at enrollment to the age of the first diagnosis of any invasive cancer, death, loss of follow-up, or mid-September 2021, whichever occurred first. We used Cox proportional hazards models adjusted for race/ethnicity with attained age as the timescale to estimate hazard ratios (HR) for DTC incidence in relation to in-utero and newborn characteristics. For the main analyses, parental ages at delivery, birth weight, birth order, and time since last pregnancy of participants’ mother were considered both categorically and continuously. Missing data were excluded from analyses of continuous variables. There was no evidence of departure from linearity for the association between continuous variables and thyroid cancer incidence when assessing with Martingale residuals. Non-proportional hazards assumptions were assessed through plots of scaled Schoenfeld residuals against attained age, and formal testing included introducing an interaction term between exposures and attained age; no evidence of violation was identified.

We conducted several sensitivity analyses including (1) considering death and the diagnosis of invasive cancer other than thyroid and non-melanoma cancers as competing risks using Fine-Gray models [23], (2) restricting the outcome to pathologically confirmed cases, (3) restricting the outcome to papillary thyroid carcinomas, (4) conducting complete case analysis by excluding individuals with missing data on a model-specific basis, and (5) calculating E-values for both the observed association estimates and the limit of the confidence interval closest to the null. The E-value is defined as the minimum strength of association that an unmeasured confounder would need to have with both the exposure and the outcome, conditional on the measured covariates, to fully explain the observed associations [24, 25]. We also conducted stratified analyses according to known thyroid cancer risk factors and potential effect modifiers, including baseline BMI, benign thyroid disease status, and socioeconomic indicators (i.e., personal attained educational level and household annual income). Tests for multiplicative interactions were conducted by including a cross-product term in the model and evaluating the F-test-based *P* value. *P* values were two-sided with an alpha of 0.05.

Data analyses were conducted using SAS 9.4, R version 4.3.1 and R studio version 2023.06.2.

## Results

The baseline descriptive statistics are provided in Table 1. The median age at baseline was 55.4 years (interquartile range, IQR, 48.9–62.1). Among 47,913 women included in the study, most participants were non-Hispanic White (*n* = 39947, 83.4%), and 30.0% (*n* = 14361) reported having a BMI of 30 or more. Approximately half of the participants had completed a bachelor’s degree or higher (*n* = 24450, 51.0%) and 33.7% reported a household annual income of $100,000 or more (*n* = 16133).

**Table 1:** Baseline characteristics of the study sample (*n* = 47,913).

Characteristic	
Age (median, interquartile range)	55.4 (48.9, 62.1)
Race/ethnicity	
Non-Hispanic White	39,947 (83.4%)
Non-Hispanic Black	4304 (9.0%)
Hispanic	2416 (5.0%)
Non-Hispanic all other races	1231 (2.6%)
Unknown	15 (<0.1%)
BMI	
<25.0	18,331 (38.3%)
25.0-29.9	15,205 (31.7%)
30.0+	14,361 (30.0%)
Unknown	16 (<0.1%)
Benign thyroid disease	10,308 (21.5%)
Personal educational level	
High school, GED, or less	7290 (15.2%)
Some college or associate or technical degree	16,161 (33.7%)
Bachelor’s degree or more	24,450 (51.0%)
Unknown	12 (<0.1%)
Annual household income	
<$50,000	12,228 (25.5%)
$50,000-$99999	19,552 (40.8%)
$100,000+	16,133 (33.7%)

*GED* general educational development.

During follow-up (median: 13.1 years, IQR 11.5–15), 239 cases of incident DTC were reported. We found a higher DTC incidence among women born to mothers with pre-pregnancy or gestational diabetes (HR = 2.36, 95%CI 0.97–5.74, 5 affected cases), and gestational hypertension or hypertension-related disorders (HR = 1.99, 95%CI 1.20–3.32, 15 affected cases), compared to those born to mothers without these conditions. Conversely, a lower DTC incidence was observed in women who were born at least two weeks before their due date (HR = 0.47, 95%CI 0.23–0.97, 8 affected cases), compared to those born within two weeks or after their due date. Compared to those with a birth weight between 2500 and 3999 g, individuals with a lighter birth weight had a lower DTC incidence (HR = 0.60, 95%CI 0.32–1.14, 10 affected cases), while those with a heavier birth weight had a higher incidence (HR = 1.50, 95%CI 0.96–2.33, 23 affected cases). For every additional kilogram in birth weight, there was a 24% increased incidence of DTC (95%CI 0.95–1.60). We found no associations of thyroid cancer incidence with other maternal, paternal, and newborn characteristics (Table 2).

**Table 2:** Association between in-utero and newborn factors and differentiated thyroid cancer incidence in the Sister Study cohort.

Characteristic		Multivariable models			
	Total cohort, *N* (%)	DTC cases, *N*	Person-years	HR^a^	95% CI^a^
**Maternal pregnancy** characteristics					
Pre-pregnancy or gestational diabetes					
Probably not/Definitely not	46,011 (96.0%)	230	562,673	1	—
Definitely/Probably	432 (0.9%)	5	5198	2.36	0.97, 5.74
Unknown	1470 (3.1%)	4	15,265	–	–
Gestational hypertension or hypertension-related disorders					
Probably not/Definitely not	39,918 (83.3%)	199	490,021	1	—
Definitely/Probably	1634 (3.4%)	16	19,753	1.99	1.20, 3.32
Unknown^b^	6361 (13.3%)	24	73,361	–	–
Morning sickness with vomiting					
Probably not/Definitely not	12,622 (26.3%)	69	157,858	1	—
Definitely/Probably	16,953 (35.4%)	94	207,073	1.07	0.78, 1.46
Unknown^b^	18,338 (38.3%)	76	218,204	–	–
Smoking during pregnancy					
Probably not/Definitely not	30,588 (63.8%)	154	373,485	1	—
Definitely/Probably	14,212 (29.7%)	72	174,538	0.99	0.74, 1.31
Unknown^b^	3113 (6.5%)	13	35,112	–	–
Household secondhand smoking during pregnancy					
Probably not/Definitely not	18,209 (38.0%)	104	224,258	1	—
Definitely/Probably	25,430 (53.1%)	122	310,669	0.86	0.66, 1.12
Unknown^b^	4274 (8.9%)	13	48,208	–	–
Time since last pregnancy					
First born	10,702 (22.3%)	57	130,433	1	—
Equal or less than 2 years	17,181 (35.9%)	88	209,459	0.92	0.66, 1.30
2–5 years	12,797 (26.7%)	66	157,276	0.91	0.64, 1.30
More than 5 years	5274 (11.0%)	20	65,010	0.66	0.40, 1.11
Unknown^b^	1959 (4.1%)	8	20,958	–	–
Time since last pregnancy (per year, continuous)^c^	45,954	174	431,744	0.97	0.91, 1.03
Age at delivery (years)					
<25 years	13,354 (27.9%)	70	160,261	1	—
25–29 years	14,194 (29.6%)	74	174,458	0.95	0.68, 1.31
30–34 years	10,862 (22.7%)	50	133,930	0.82	0.57, 1.19
35+	8545 (17.8%)	41	104,668	0.86	0.58, 1.27
Unknown^b^	958 (2.0%)	4	9817	-	-
Age at delivery (per year, continuous)	46,955	235	573,318	0.99	0.97, 1.01
**Paternal pregnancy characteristics**					
Smoking during 3 months before conception					
Probably not/Definitely not	14,835 (31.0%)	78	183,156	1	—
Definitely/Probably	29,089 (60.7%)	145	354,729	0.99	0.75, 1.30
Unknown^b^	3989 (8.3%)	16	45,250	–	–
Age at delivery					
<25 years	6687 (14.0%)	34	79,488	1	—
Between 25 and 29 years	12,802 (26.7%)	62	157,065	0.9	0.60, 1.37
Between 30 and 34 years	12,174 (25.4%)	63	150,351	0.95	0.63, 1.44
35+ years	15,032 (31.4%)	74	183,618	0.91	0.61, 1.37
Unknown^b^	1218 (2.5%)	6	12,613	–	–
Age at delivery (per year, continuous)	46,695	233	570,522	1.00	0.98, 1.02
**Birth and infancy characteristics**					
Birth weight (g)					
<2500	3562 (7.4%)	10	42,933	0.6	0.32, 1.14
2500–3999	28,273 (59.0%)	140	349,938	1	—
4000+	3150 (6.6%)	23	38,632	1.50	0.96, 2.33
Unknown^b^	12,928 (27.0%)	66	151,632	-	-
Birth weight (per kg, continuous)	34,674	172	427,789	1.24	0.95, 1.60
Birth order					
First born	10,702 (22.3%)	57	130,433	1	—
Second born	12,078 (25.2%)	61	148,388	0.91	0.63, 1.30
Third born	9396 (19.6%)	43	115,772	0.81	0.54, 1.21
Fourth born or more	13,778 (28.8%)	70	167,573	0.9	0.63, 1.30
Unknown^b^	1959 (4.1%)	8	20,970	-	-
Birth order (per birth, continuous)	45,954	231	562,165	1.01	0.95, 1.08
Multiple birth					
Single birth	45,664 (95.3%)	230	557,281	1	—
Multiple birth	1493 (3.1%)	6	18,210	0.8	0.36, 1.80
Unknown^b^	756 (1.6%)	3	7644	–	–
Gestational age at birth					
Born post-term or <2 weeks before due date	18,956 (39.6%)	116	237,133	1	—
Born 2 or more weeks before the due date	2778 (5.8%)	8	34,695	0.47	0.23, 0.97
Unknown^b^	26,179 (54.6%)	115	311,308	–	–
Ever breastfed					
Probably not/Definitely not	22,246 (46.4%)	119	274,855	1	—
Definitely/Probably	21,310 (44.5%)	104	259,040	0.97	0.74, 1.27
<6 weeks	2241 (4.7%)	15	28,508	1.22	0.71, 2.08
6 weeks–3 months	3175 (6.6%)	13	39,500	0.76	0.43, 1.35
4–6 months	2933 (6.1%)	16	36,075	1.05	0.62, 1.76
>6 months	4850 (10.1%)	21	58,399	0.86	0.53, 1.38
Unknown duration^b^	8111 (16.9%)	39	96,558	–	–
Unknown^b^	4357 (9.1%)	16	49,240	–	–

*HR* Hazard Ratio, *CI* Confidence Interval.

^a^Multivariable models used attained age as the timescale and were adjusted for race/ethnicity.

^b^Results for “Unknown” categories are not shown.

^c^Individuals who were first born were excluded.

Because of the potential co-occurrence of gestational complications (e.g., gestational diabetes and hypertension) and associations with preterm delivery and small-for-gestational-age births [26], we evaluated the independent associations of pregnancy complications, gestational length at birth, and birth weight in mutually adjusted models. The HRs were similar, albeit slightly weaker for pre-pregnancy and gestational diabetes and birth weight (Table 3). The association for birth weight did not meaningfully change after excluding participants who were born at least two weeks before the due date or part of a multiple birth (167 cases, HR per kg = 1.16, 95% CI 0.87–1.56). The results were also consistent after further excluding those whose mothers had pre-pregnancy or gestational diabetes and gestational hypertension or hypertension-related disorders (152 cases, HR per kg = 1.13, 95% CI 0.83–1.54) (Supplementary Table 1). No significant interactions were observed for birth weight and DTC risk by baseline BMI, benign thyroid disease, or household annual income, although the association was strongest for women in the lowest category of education (high school education, GED diploma, or less, 18 exposed cases, HR = 2.61 for every additional kilogram, 95% CI 1.22–5.59) (Supplementary Table 2).

**Table 3:** Mutually-adjusted associations between differentiated thyroid cancer risk factors.

Characteristic	DTC cases, *N*	Person-years	HR^a^	95% CI^a^
Pre-pregnancy or gestational diabetes				
Probably not/Definitely not	230	562,673	1	—
Definitely/Probably	5	5198	1.89	0.76, 4.67
Unknown^b^	4	15,265	–	–
Gestational hypertension or hypertension-related disorders				
Probably not/Definitely not	199	490,021	1	—
Definitely/Probably	16	19,753	2.00	1.19, 3.35
Unknown^b^	24	73,361	–	–
Gestational age at birth				
Born post-term or up to 2 weeks before the due date	116	237,133	1	—
Born at least 2 weeks before the due date	8	34,695	0.54	0.26, 1.15
Unknown^b^	115	311,308	–	–
Birth weight (g)				
<2500	10	42,933	0.7	0.36, 1.36
2500–3999	140	349,938	1	—
4000+	23	38,632	1.43	0.92, 2.24
Unknown^b^	66	151,632	–	–

*HR* Hazard Ratio, *CI* Confidence Interval.

^a^Multivariable models used attained age as the timescale and were adjusted for race/ethnicity.

^b^Results for “Unknown” categories are not shown.

The interpretation of the findings did not change when accounting for deaths and other cancer diagnoses as competing risks or when considering only medically confirmed thyroid cancer, papillary thyroid cancer cases, or complete case analysis (Supplementary Table 3). The E-values for the associations of gestational hypertension or hypertension-related disorders and being born at least two weeks before the due date, and birth weight less than 2500 g were 3.40, and 3.65, respectively.

## Discussion

Due to a paucity of data, few studies have examined early life exposures in relation to thyroid cancer risk later in life. Using data from a large nationwide U.S. cohort of women, the current study revealed notable associations for several in-utero and newborn factors, including higher birth weight and maternal metabolic complications (e.g., pre-pregnancy and gestational diabetes, gestational hypertension or hypertension-related disorders). Conversely, being born at least two weeks before the due date was associated with a lower incidence of DTC.

The magnitude of the association for birth weight (24% higher DTC risk for each additional kilogram, 95% CI 0.95–1.60) is consistent with results from previous studies suggesting increased risks between 14 and 30% per kilogram [17, 19]. Studies on early life exposures and childhood thyroid cancer incidence (age at diagnosis <20 years) have yielded less consistent results, with two studies reporting positive associations with risks varying between 11 and 12% per 500 grams [18, 27], while one study showed no association [28]. However, considering that the etiology of childhood thyroid cancer may be different from adulthood thyroid cancer, findings from these latter studies may not be directly comparable to ours.

Only one prior study extensively examined the associations of maternal pregnancy complications and DTC risk among adult offspring [17]. In a registry-based study combining data from four Nordic countries (mean age at diagnosis: 27.5 years), Kitahara et al. found increased incidence of thyroid cancer associated with maternal benign thyroid conditions (OR ranging from 3.3 to 67.4), maternal postpartum hemorrhage (132 exposed cases, OR = 1.28, 95%CI 1.06-1.55), pre-pregnancy diabetes (15 exposed cases, OR = 1.69, 95%CI 0.98–2.93), and congenital hypothyroidism (5 exposed cases, OR = 4·55, 95% CI 1.58-13.1). However, no associations were observed for gestational diabetes or pre-pregnancy or gestational hypertension [17].

Birth weight and maternal complications have been suggested to influence BMI in adolescence and adulthood [29–31], as well as adult benign thyroid disease [32]. Thus, associations of these perinatal factors with DTC incidence may be mediated by these downstream factors. However, this hypothesis was not supported by our data, as positive associations of perinatal exposures and DTC incidence persisted after stratifying by adulthood exposures. This suggests that biological factors contributing to thyroid cancer development may originate at a very young age.

Birth weight, preterm delivery, and maternal complications are closely associated factors [26]. In the current study, excluding women who were born at least two weeks before the due date or who were part of a multiple birth did not change the results for birth weight, suggesting that the association for birth weight remains independent of preterm birth. The associations between birth weight and pre-pregnancy and gestational diabetes with DTC were somewhat attenuated after adjusting for gestational hypertension or pre-eclampsia, indicating the presence of both shared and distinct underlying mechanisms. Both higher birth weight and gestational diabetes may serve as markers for aspects of the fetal environment, such as increased levels of growth factors [33] or insulin receptor activation leading to IGF-I overproduction [34]. Epidemiologic and molecular studies support a possible mediating role of IGF-I, IGF binding protein, and deregulation of the IGF axis in thyroid carcinogenesis [15, 35]. Furthermore, higher birth weight may indicate elevated levels of fetal thyroid hormones [36], with longitudinal studies suggesting that even within the clinically normal range, low levels of thyroid-stimulating hormone and high levels of thyroid hormones in adults are associated with thyroid cancer incidence [37]. On the other hand, gestational diabetes may contribute to thyroid cancer development through the effects of hyperglycemia and hyperinsulinemia on epigenetic changes in the offspring [38].

Maternal metabolic complications may also result from exposure to EDCs [39], which have been implicated in thyroid cancer development in adults [10]. While most EDC exposures do not appear to significantly affect fetal thyroid function [40], certain chemicals—such as polybrominated diphenyl ethers, per- and polyfluoroalkyl substances—can potentially impair thyroid hormone enzyme activity, reduce iodine availability to the fetus, and disrupt fetal thyroid function [13, 41]. In the Sister Study, the rarity of in-utero exposure to endocrine disruptors, such as diethylstilbestrol, and the lack of data on maternal and fetal exposures to EDCs before and during pregnancy [42] in this study precluded a direct evaluation of their effects on thyroid cancer risk in offspring. Given the lack of prior studies on this association, future studies should include detailed assessments of both maternal and fetal EDC exposure and metabolic conditions.

Strengths of this study include the large sample size, long follow-up, and wide range of in-utero and birth characteristics. While detection bias is a significant consideration in epidemiological studies on thyroid cancer incidence [2], it is unlikely to explain the notable associations in our study, as there is no apparent connection between the in-utero and newborn factors examined in our study and access to thyroid or cancer screening in adulthood. Our analysis also included an evaluation of potential factors that could influence the likelihood of incidental detection of thyroid cancer, such as household annual income and educational levels; the strength of the associations did not differ substantially by these factors.

The current study has several limitations. As the Sister Study recruited only women with a family history of breast cancer, the results may not be generalizable to men or to women without a family history of breast cancer. Self-reported early-life exposures may be prone to misclassification bias, potentially with varying degree of validity [43–45], although it is unlikely that report of perinatal characteristics varied by future case status; this type of bias is expected to drive associations toward the null. Although the Sister Study provided phone cards to encourage participants to contact their mothers or other relatives, whether participants contacted relatives or consulted records before reporting early-life events is unclear. Despite the large size of this cohort, there were small case numbers corresponding with some exposures of interest, which limited the statistical power of our study and our ability to test for interactions between different in-utero and newborn factors. We lacked information on maternal thyroid status, pre-pregnancy hypertension, pre-pregnancy and pregnancy BMI, and maternal weight gain during pregnancy. High maternal pre-pregnancy BMI and excessive weight gain during pregnancy have been suggested to be predictors for metabolic syndrome and obesity in the offspring [46, 47], thus potentially influencing the study results. We did not account for some well-established (e.g., exposure to ionizing radiation in childhood) or possible risk factors (e.g., iodine deficiency) of thyroid cancer, but these factors were unlikely to have been strong confounders as they are not likely to be associated with other in-utero and newborn exposures. The high E-values in analyses concerning maternal complications indicated that HRs of at least 3 for the unmeasured confounder and DTC incidence would be necessary to fully explain the observed associations.

In conclusion, our findings contribute to a growing body of evidence that in-utero exposures related to maternal metabolic abnormalities may influence thyroid cancer risk later in life, suggesting a potential target in the primary prevention of DTC. Further research using objective exposure information collected from birth and medical records or biological measurements at the time of pregnancy or birth are needed to confirm our findings while minimizing the potential for exposure misclassification and provide additional mechanistic insights.

## Supplementary information

Supplementary table 1: Association between birth weight and differentiated thyroid cancer incidence after excluding (1) participants who were born 2 or more weeks before the due date or those who were

Supplementary Table 2: Association between birth weight (per kg, continuous) and differentiated thyroid cancer incidence in the Sister Study cohort by baseline BMI, benign thyroid disease, personal ed

Supplementary table 3: Association between in-utero and newborn factors and differentiated thyroid cancer incidence in the Sister Study participants: sensitivity analyses

## Data Availability

Data used in this article are available as described on the Sister Study website (The Sister Study: Collaborations and Data Requests, nih.gov; https://sisterstudy.niehs.nih.gov/English/cohort-over.htm; https://sisterstudy.niehs.nih.gov/English/enroll-data.htm#SAQS) or by request via the Sister Study tracking and review system (www.sisterstudystars.org; registration required).

## Code availability

Computing code is available at: https://github.com/tvtrinh-tran/In-utero_factors_Thyroid_Cancer_Incidence.
