# Supplementary material for: In-utero and newborn factors and thyroid cancer incidence in adult women in the Sister Study cohort
Source: Br J Cancer. 2025 Apr 9;132(11):1056–63. doi: 10.1038/s41416-025-03004-6 (PMC12119869; doi:10.1038/s41416-025-03004-6)
Supplement: Supplementary file 1 — Supplementary table 1: Association between birth weight and differentiated thyroid cancer incidence after excluding (1) participants who were born 2 or more weeks before the due date or those who were [file 41416_2025_3004_MOESM1_ESM.docx]

**Supplementary table 1: Association between birth weight and differentiated thyroid cancer incidence after excluding (1) participants who were born 2 or more weeks before the due date or those who were part of a multiple pregnancy (N = 3,806), and (2) additionally excluding those whose mothers had pre-pregnancy or gestational diabetes and gestational hypertension or hypertension-related disorders (N = 5,431)**

| **Characteristic** |  | **Exclusion 1** | | | | **Exclusion 2** | | | |
| --- | --- | --- | --- | --- | --- | --- | --- | --- | --- |
|  | **N (%)** | **DTC cases, N** | **Person-years** | **HR** | **95%CI** | **DTC cases, N** | **Person-years** | **HR** | **95%CI** |
| Birth weight (g) |  |  |  |  |  |  |  |  |  |
| < 2500 | 1,858 (4.2%) | 6 | 22,099 | 0.69 | 0.31, 1.57 | 6 | 21,075 | 0.74 | 0.33, 1.69 |
| 2500 - 3999 | 26,659 (60.4%) | 134 | 329,615 | 1 | — | 127 | 317,828 | 1.00 | — |
| 4000+ | 3,109 (7.0%) | 23 | 38,133 | 1.49 | 0.96, 2.32 | 19 | 35,323 | 1.35 | 0.83, 2.19 |
| Unknown^1^ | 12,481 (28.3%) | 64 | 146,290 | - | - | 59 | 142,346 | - | - |
| Birth weight (per kg, continuous) | 31,485 | 163 | 388,231 | 1.16 | 0.87, 1.56 | 152 | 372,691 | 1.13 | 0.83, 1.54 |
| HR = Hazard Ratio, CI = Confidence Interval. Multivariable models used attained age as the timescale and were adjusted for self-reported race/ethnicity | | | | | | | | | |
| Exclusion 1: Excluding participants who were born 2 or more weeks before the due date or those who were part of a multiple pregnancy | | | | | | | | | |
| Exclusion 2: Excluding participants who were born 2 or more weeks before the due date or those who were part of a multiple pregnancy and participants whose mothers had pre-pregnancy or gestational diabetes, gestational hypertension, or hypertension-related disorders | | | | | | | | | |
| *^1^* Results for “Unknown” categories are not shown | | | | | | | | | |
