# Supplementary material for: In-utero and newborn factors and thyroid cancer incidence in adult women in the Sister Study cohort
Source: Br J Cancer. 2025 Apr 9;132(11):1056–63. doi: 10.1038/s41416-025-03004-6 (PMC12119869; doi:10.1038/s41416-025-03004-6)
Supplement: Supplementary file 2 — Supplementary Table 2: Association between birth weight (per kg, continuous) and differentiated thyroid cancer incidence in the Sister Study cohort by baseline BMI, benign thyroid disease, personal ed [file 41416_2025_3004_MOESM2_ESM.docx]

**Supplementary Table 2: Association between birth weight (per kg, continuous) and differentiated thyroid cancer incidence in the Sister Study cohort by baseline BMI, benign thyroid disease, personal educational level, and annual household income (N = 34674)**

| **Characteristic^1^** | **DTC cases, N** | **Person-years** | **HR^2^** | **95% CI^2^** |
| --- | --- | --- | --- | --- |
| **BMI (kg/m^2^)** |  |  |  |  |
| <25 | 63 | 175,075 | 0.89 | 0.57, 1.39 |
| 25.0-29.9 | 49 | 132,821 | 1.35 | 0.84, 2.18 |
| 30.0+ | 60 | 119,777 | 1.49 | 0.98, 2.28 |
| *p-interaction* |  |  | 0.39 |  |
| **Benign thyroid disease** |  |  |  |  |
| No | 131 | 337,775 | 1.15 | 0.85, 1.55 |
| Yes | 41 | 90,015 | 1.58 | 0.93, 2.67 |
| *p-interaction* |  |  | 0.32 |  |
| **Personal educational level** |  |  |  |  |
| High school or GED or less | 18 | 58,058 | 2.61 | 1.22, 5.59 |
| Some college or associate or technical degree | 60 | 140,071 | 1.12 | 0.73, 1.74 |
| Bachelor’s degree or higher | 94 | 229,544 | 1.12 | 0.78, 1.61 |
| *p-interaction* |  |  | 0.29 |  |
| **Annual household income** |  |  |  |  |
| <$50000 | 44 | 95,923 | 1.35 | 0.83, 2.21 |
| $50000-$99999 | 69 | 177,923 | 1.17 | 0.78, 1.75 |
| $100000+ | 59 | 153,944 | 1.22 | 0.76, 1.95 |
| *p-interaction* |  |  | 0.90 |  |
| HR = Hazard Ratio, CI = Confidence Interval  ^1^ Results for “Unknown” categories of are not shown  *^2^* Multivariable models used attained age as the timescale and were adjusted for self-reported race/ethnicity | | | | |
