# Supplementary material for: In-utero and newborn factors and thyroid cancer incidence in adult women in the Sister Study cohort
Source: Br J Cancer. 2025 Apr 9;132(11):1056–63. doi: 10.1038/s41416-025-03004-6 (PMC12119869; doi:10.1038/s41416-025-03004-6)
Supplement: Supplementary file 3 — Supplementary table 3: Association between in-utero and newborn factors and differentiated thyroid cancer incidence in the Sister Study participants: sensitivity analyses [file 41416_2025_3004_MOESM3_ESM.docx]

## **Supplementary table 3: Association between in-utero and newborn factors and differentiated thyroid cancer incidence in the Sister Study participants: sensitivity analyses**

| **Characteristic** | **Competing risks** | | | **Medically confirmed cases only** | | | **Papillary thyroid cancer only** | | | **Complete case analysis** | | | **E-values** | | |
| --- | --- | --- | --- | --- | --- | --- | --- | --- | --- | --- | --- | --- | --- | --- | --- |
|  | **Number of cases** | **HR***^1^* | **95% CI***^1^* | **Number of cases** | **HR***^1^* | **95% CI***^1^* | **Number of cases** | **HR***^1^* | **95% CI***^1^* | **Number of cases** | **HR***^1^* | **95% CI***^1^* | **E-values for HR** | **E-values for CI^2^** | |
| **Maternal pregnancy characteristics** |  |  |  |  |  |  |  |  |  |  |  |  |  |  | |
| Pre-pregnancy or gestational diabetes |  |  |  |  |  |  |  |  |  |  |  |  |  |  | |
| Probably not/Definitely not | 230 | 1 | — | 168 | 1 | — | 157 | 1 | — | 230 | 1 | — |  |  | |
| Definitely/Probably | 5 | 2.53 | 1.05, 6.12 | 5 | 3.3 | 1.35, 8.05 | 5 | 3.56 | 1.46, 8.70 | 5 | 2.38 | 0.98, 5.79 | 4.16 | 1 | |
| Unknown^3^ | 4 | - | - | 2 | - | - | 2 | - | - | 0 |  |  | - | - | |
| Gestational hypertension or hypertension-related disorders |  |  |  |  |  |  |  |  |  |  |  |  |  |  | |
| Probably not/Definitely not | 199 | 1 | — | 145 | 1 | — | 138 | 1 | — | 199 | 1 | — |  |  | |
| Definitely/Probably | 16 | 1.94 | 1.17, 3.23 | 12 | 2.07 | 1.15, 3.73 | 9 | 1.63 | 0.83, 3.21 | 16 | 2 | 1.20, 3.33 | 3.4 | 1.68 | |
| Unknown^3^ | 24 | - | - | 18 | - | - | 17 | - | - | 0 |  |  | - | - | |
| **Birth and infancy characteristics** |  |  |  |  |  |  |  |  |  |  |  |  |  |  | |
| Birth weight (g) |  |  |  |  |  |  |  |  |  |  |  |  |  |  | |
| < 2500 | 10 | 0.59 | 0.31, 1.13 | 6 | 0.48 | 0.21, 1.09 | 5 | 0.42 | 0.17, 1.03 | 10 | 0.6 | 0.32, 1.15 | 2.72 | 1 | |
| Between 2500 and 3999 | 140 | 1 | — | 106 | 1 | — | 101 | 1 | — | 140 | 1 | — |  |  | |
| 4000+ | 23 | 1.54 | 0.99, 2.39 | 16 | 1.39 | 0.82, 2.35 | 14 | 1.28 | 0.73, 2.24 | 23 | 1.5 | 0.96, 2.33 | 2.36 | 1 | |
| Unknown^3^ | 66 | - | - | 47 | - | - | 44 | - | - | 0 |  |  | - | - | |
| Birth weight (per kg, continuous) | 172 | 1.26 | 0.97, 1.63 | 127 | 1.34 | 0.99, 1.82 | 119 | 1.37 | 1.00, 1.87 | 172 | 1.24 | 0.95, 1.60 | 1.78 | 1 | |
| Gestational age at birth |  |  |  |  |  |  |  |  |  |  |  |  |  |  | |
| Born post-term or less than 2 weeks before due date | 116 | 1 | — | 91 | 1 | — | 84 | 1 | — | 116 | 1 | — |  |  | |
| Born at least 2 weeks before due date | 8 | 0.48 | 0.23, 0.98 | 6 | 0.45 | 0.20, 1.03 | 5 | 0.41 | 0.17, 1.01 | 8 | 0.48 | 0.23, 0.97 | 3.65 | 1.22 | |
| Unknown^3^ | 115 | - | - | 78 | - | - | 75 | - | - | 0 |  |  | - | - | |
| HR = Hazard Ratio, CI = Confidence Interval. The risk estimates are shown when there were at least 5 cases.  *^1^* Multivariable models used attained age as the timescale and were adjusted for self-reported race/ethnicity  *^2^* E-values for the limit of the confidence interval closest to the null  ^3^ Results for “Unknown” categories of are not shown | | | | | | | | | | | | | | |  |
